# Digital Health Readiness: Making Digital Health Care More Inclusive

**DOI:** 10.2196/58035

**Published:** 2024-10-09

**Authors:** Timothy Bober, Bruce L Rollman, Steven Handler, Andrew Watson, Lyndsay A Nelson, Julie Faieta, Ann-Marie Rosland

**Affiliations:** 1 Division of General Internal Medicine University of Pittsburgh School of Medicine Pittsburgh, PA United States; 2 Center for Research on Health Care University of Pittsburgh Pittsburgh, PA United States; 3 Center for Behavioral Health, Media, and Technology University of Pittsburgh Department of Medicine Pittsburgh, PA United States; 4 Center for Health Equity Research and Promotion VA Pittsburgh Healthcare System Pittsburgh, PA United States; 5 Division of Geriatric Medicine University of Pittsburgh Pittsburgh, PA United States; 6 Department of Rehab Science and Technology University of Pittsburgh Pittsburgh, PA United States; 7 Technology Enhancing Cognition and Health – Geriatrics Research Education and Clinical Center VA Pittsburgh Healthcare System Pittsburgh, PA United States; 8 Department of Surgery University of Pittsburgh Pittsburgh, PA United States; 9 UPMC Health Plan Pittsburgh, PA United States; 10 UPMC Enterprises Pittsburgh, PA United States; 11 Division of General Internal Medicine and Public Health Department of Medicine Vanderbilt University Medical Center Nashvillie, TN United States

**Keywords:** digital health, digital health literacy, informatics, digital disparities, digital health readiness, inclusivity, digital health tool, literacy, patient support, health system

## Abstract

This paper proposes an approach to assess digital health readiness in clinical settings to understand how prepared, experienced, and equipped individual people are to participate in digital health activities. Existing digital health literacy and telehealth prediction tools exist but do not assess technological aptitude for particular tasks or incorporate available electronic health record data to improve efficiency and efficacy. As such, we propose a multidomain digital health readiness assessment that incorporates a person’s stated goals and motivations for use of digital health, a focused digital health literacy assessment, passively collected data from the electronic health record, and a focused aptitude assessment for critical skills needed to achieve a person’s goals. This combination of elements should allow for easy integration into clinical workflows and make the assessment as actionable as possible for health care providers and in-clinic digital health navigators. Digital health readiness profiles could be used to match individuals with support interventions to promote the use of digital tools like telehealth, mobile apps, and remote monitoring, especially for those who are motivated but do not have adequate experience. Moreover, while effective and holistic digital health readiness assessments could contribute to increased use and greater equity in digital health engagement, they must also be designed with inclusivity in mind to avoid worsening known disparities in digital health care.

## Introduction

The use of digital tools for health care—including video visits, patient portals, mobile apps, and remote monitors—has risen exponentially over the last decade and become more essential for care access during and after the COVID-19 pandemic [1,2]. Patients using digital health tools have been shown to have better outcomes in managing many outpatient health conditions, including diabetes [3,4], anxiety and mood disorders [5], hypertension [6], and chronic pain [7]. Still, despite their growing incorporation into health care and potential to improve health outcomes, many who could benefit from these tools are not using them [1,2,8,9]. If health systems can develop approaches to close this gap with innovative and tailored pathways to digital health care, they could improve access, inclusivity, and outcomes.

**Figure 1:**
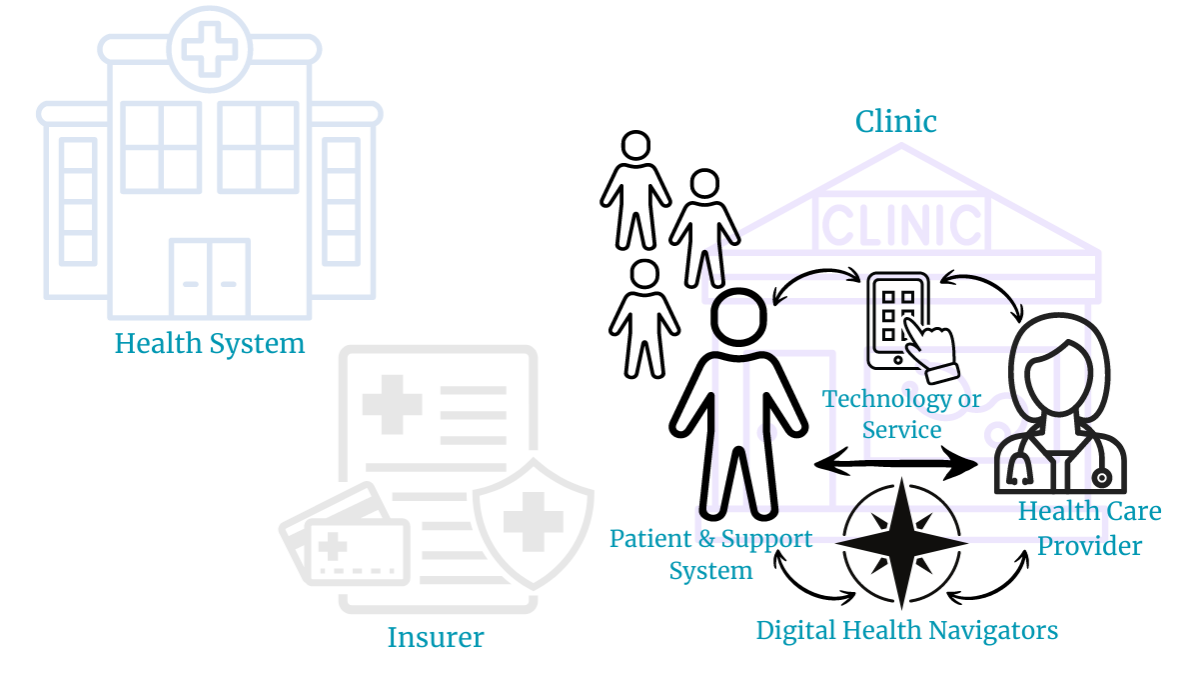
Health care system components of a proposed digital health readiness ecosystem. These are the possible health care nodes of a digital health readiness ecosystem—all of which impact digital health use—including the technology or service, patients and their support system, health care providers, digital health navigators, insurers, and clinics and health systems.

Prior approaches to increase digital health engagement focused on several domains, including such logistical factors as broadband internet access [10], access to smartphones, and the ability of individuals to use technology to participate in health care and understand their health (ie, digital health literacy) [11]. Initial assessments of digital health literacy in the mid-2000s focused on the ability to use the internet, but they have since expanded to encompass smartphones, mobile apps, and social media [12,13]. As a construct, digital health literacy has also grown to reflect multiple domains of health technology use, including personal aspects like prior experiences, digital self-efficacy, motivation to use digital health, and access to technology [14]. The evolution of these assessments reflects changes in the technological environment but also demonstrates the multifaceted nature of digital health literacy overall. Future approaches to facilitating further equitable growth of digital health could consider the ecosystem of factors that drive engagement with these tools. General health literacy is increasingly understood as a relational concept in which patients and health care providers (HCPs) balance their skills and abilities against the demands of health care systems [15,16]. Digital health readiness for individual patients exists within similar contexts and is impacted by the technological tools themselves (particularly the demands that they place on patients), the HCPs prescribing and monitoring their use, the clinics and digital health navigation services where technological instruction occurs, the health systems and their approach toward digital health implementation, and the insurers that control coverage of these services and tools (Figure 1). In this paper, we review current digital health literacy measures to assess and predict a person’s ability to engage with digital health, discuss their relative strengths and weaknesses, and describe our holistic vision for health care systems to assess digital health readiness efficiently with health record data.

Multidomain digital health readiness assessments could create a phenotype for each patient representing how prepared, experienced, and equipped they are to use a particular digital health tool at a certain point in time [17]. Prior studies have established approaches to understand readiness within health systems (ie, how prepared and experienced a system is for digital care implementation) [18], within individual health care facilities [19], and among health professionals themselves [20]. Approaches for comprehensively defining and assessing individual-level digital health readiness could become central to health system and payor operations, as signaled by the Center for Medicare and Medicaid Services (CMS) mandate that Medicare Advantage organizations offer “digital health education” for telehealth to their members [21].

Creating effective and holistic digital health readiness assessments could contribute to increased use of and access to these tools among patients and their families. In this paper, we focus only on assessing individual, patient-level digital health readiness, but we acknowledge that this construct can be applied to any node within the digital health readiness ecosystem, as noted above and in Figure 1.

## Strengths and Weaknesses of Current Digital Health Readiness Measures

Current methods to assess digital health readiness have several strengths and weaknesses.

One strength of these measures is that they assess relevant aspects of digital health participation and are often short enough to be incorporated into clinical practice; however, these measures assess personal attitudes alone without considering technological aptitude. For example, the eHealth Literacy Scale (eHEALS) is the most cited digital health literacy measure and focuses on assessing a person’s attitudes, confidence, and subjective skill level in using internet search engines and evaluating online information, yet it does not assess the experience needed for smartphones and wearable monitors or address such structural factors as device access (either through personal ownership or sharing) [13]. Newer measures such as the Digital Health Care Literacy Scale do capture skills for using and troubleshooting mobile apps and videoconferencing apps in a brief manner that is primed for clinical settings, but they also do not assess technical aptitude or device access [22]. For digital health readiness assessments to be useful in the clinical operations of health systems, they should have an aptitude assessment to stratify individuals into levels with matched support interventions. Additionally, research will be needed on what demonstrated skills are most important for a particular care modality (like a video visit versus wearing a remote monitor).

More thorough digital health readiness assessments cover many relevant aspects of the digital health care experience; however, they may be logistically challenging to administer in clinical settings. For instance, the recent Digital Health Readiness Questionnaire (from 2023) gathers a more detailed assessment of a person’s experiences with digital health by asking about their skills, digital literacy, digital health literacy, device use, and learnability, but its 20 items might be cumbersome to administer in a busy primary care setting, do not assess actual aptitude, and do not include questions about device or internet access [23]. Even more robust assessments, including the eHealth Assessment Toolkit [17] and eHealth Literacy Questionnaire [24], are validated and available, though their comprehensiveness also likely makes them unwieldy for application in clinical settings. For example, the eHealth Assessment Toolkit [17] has 44 questions encompassing 7 different tools for digital health care.

One strength of contemporary digital health readiness measures is that they are grounded in updated theoretical constructs of digital health equity that aim to improve engagement with populations facing health disparities and reflect our current technological environment.

The framework for digital health equity augmented the National Institute on Minority Health and Health Disparities research framework by adding individual, interpersonal, community, and societal aspects of the digital environment and patient experience [14]. Previously elaborated digital health readiness research strategies like those from Lyles et al [25] and Jaworski et al [26] were built on components such as “access, motivation and trust, and digital health literacy” that are also fundamental for boosting digital health engagement. Despite being published relatively recently, these frameworks are widely cited and are being incorporated into wide-ranging fields, including behavioral health research, addiction medicine, and cardiovascular medicine—among others [27,28]. While these updated constructs reflect the current experiences of being a digital health care user, they will also likely need to be updated over time to match the dynamic nature of digital health innovation and remain relevant in the frantic pace of clinical care. Moreover, as seen in the following scenario, approaches to digital health readiness will need to be agile and adaptable to meet the unique needs of each individual.

## Scenario 1: Digital Health Readiness and Wearable Health Monitors

This hypothetical patient scenario (Textbox 1) reflects the challenges of applying individual digital health readiness assessments and how clinical teams could be responsive to each person’s unique needs.

Ms T’s case demonstrates the importance of aptitude testing (eg, prompting a user to show an instructor how they might use a phone app) and how a care team might adjust a digital health care modality to best meet the needs of a patient.

Another weakness of current digital health literacy and readiness measures is that they do not integrate passively collected data from the electronic health record to improve efficiency and efficacy. Using available metrics—such as a visualized breakdown of previous in-person care, completed video visits, completed phone visits, and patient portal use— can increase the efficiency of digital health readiness assessments and portray a person’s actual care use compared with their stated goals. Examples include the Telemedicine ImPACT Score [29] and EpicCare Video Visit Technical Risk Score [30], which use data on the number of prior completed video visits and portal messages sent to forecast future digital engagement without the need to administer a questionnaire. These data seamlessly contribute information about an individual’s digital determinants of health—that is, the larger social, personal, and structural barriers that impact digital health engagement [14,31]—and could focus on particular factors that are most predictive of certain tasks (like completing a video visit) [32-34]. Looking at a person’s health record data in a digital health readiness profile, in-clinic technology navigators may find that a person has no broadband access or internet experience and recommend in-person care over virtual care until these factors are addressed. Passive health record data could refine in-person and digital care delivery so that patients are accessing resources in a way that matches their personal situations.

The essential elements needed for comprehensive and practical digital health readiness assessments will include aptitude testing, in addition to evaluating attitudes toward technology, customizing skill assessment to address emerging technologies, and incorporating passively collected health system data. Existing digital health literacy screening metrics and digital health prediction tools each have strengths that could create a more comprehensive profile of a person’s prior technological experience and could be adapted to the use of new technologies over time.

**Hypothetical patient scenario 1.**

Ms T is a woman aged 63 years with a laptop computer and a smartphone who regularly searches for health information on the internet. Ms T qualifies for a continuous glucose monitor (CGM) to track her blood sugars; however, the device typically downloads data to a smartphone for users to view their trends. She has nerve damage from diabetes that limits her ability to navigate smartphone screens, but she is able to use computer keyboards without issue. Once the CGM is ordered, the diabetes education team asks her to bring whichever devices she most commonly uses to her CGM training session. During her visit, the diabetes nurse educator evaluates her for digital health literacy using the 3-item Digital Health Care Literacy Scale and feels that she is prepared to use the CGM interface. After the educator downloads the CGM app on her smartphone, Ms T is prompted to sign in and create an account. Immediately, the staff notices that she has issues navigating the smartphone interface. Pivoting to make the technology more usable for her, they set up the CGM application on her laptop so that she can view her blood sugar trends more easily.

## Making Digital Health Readiness Assessments Practical and Efficient for Clinical Settings

**Figure 2:**
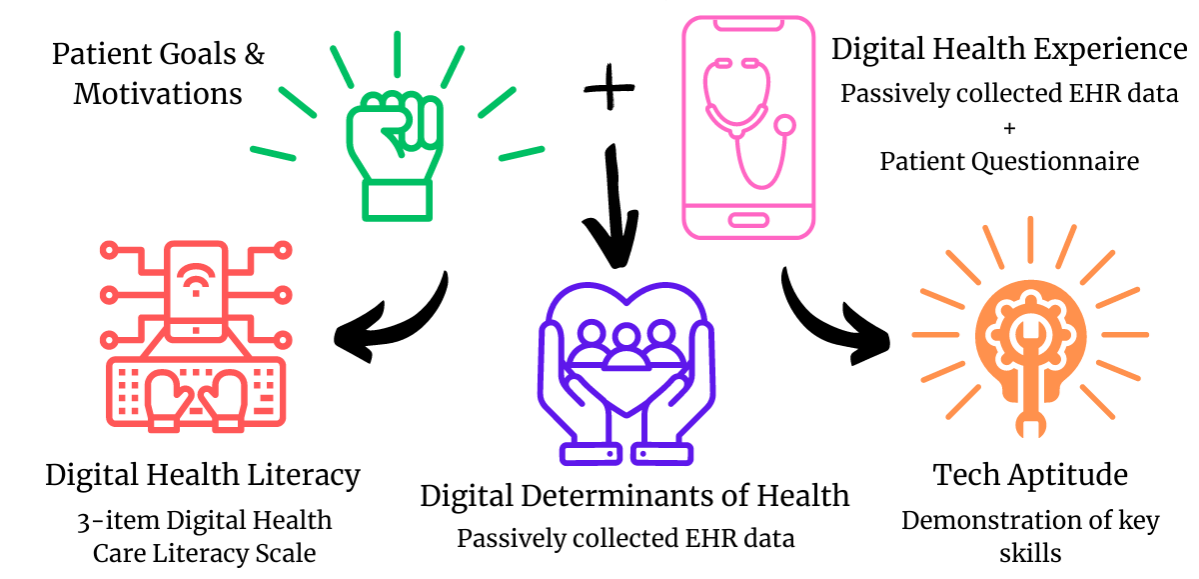
The proposed components of a holistic digital health readiness assessment based on the thoughts of the authors and the digital health equity framework of Richardson et al [14].

We envision a holistic digital heath readiness assessment that will enable health systems to deliver targeted support to those who need it most and close gaps in use. Similar to the Conversational Health Literacy Assessment Tool (CHAT), which is designed to assess multiple dimensions of a person’s health literacy in health care settings, digital health readiness assessments could be designed to provide a more comprehensive and pragmatic picture of a patient’s digital health strengths and obstacles [35]. In particular, the Health Promotion Barriers and Support, Health Information Access and Comprehension, and Current Health Behaviors domains from the CHAT could be adapted to a digital health context. Digital health readiness assessments could begin with questions about personal goals for health technology use and prior digital health experience, followed by focused aptitude testing for a particular digital health tool or goal, a brief digital health literacy assessment, and visualization of that person’s health systems data to probe into their digital determinants of health. Figure 2 reflects the proposed elements of an individual digital health readiness profile that would allow HCPs and care navigators to understand a person’s digital phenotype and act to meet their unique needs. The components of Figure 2 [14] represent our thoughts on ways to address the strengths and weaknesses outlined above and were informed by the framework for digital health equity [14]. This multi-domain approach would incorporate patient-reported data with passive data from health systems and payors to make responses more relevant and able to be added to busy clinical workflows. The key difference from existing digital health literacy assessments is the incorporation of a focused aptitude test assessment (such as having a patient show how they use a mobile app for 1-2 minutes) and the integration of passively collected clinical data. These aspects would make digital health readiness phenotyping more efficient, systematic, and, hopefully, effective for clinical settings.

As technology evolves and alters the required skills to participate in modern health care, digital health readiness assessments will need to grow in kind to reflect these skills. Ideally, the collection of inputs will differ for specific tasks. For example, completing a video visit may involve downloading a mobile app, registering an account, checking in online, and signing in to the appointment. In contrast, registering for a patient portal may involve only some of these steps. Domain-specific digital health readiness assessments could make the assessment most relevant to patients and their goals. The following fictional vignette shows how digital health readiness assessments could be tailored to help patients complete a specific task—such as how to log on to and complete a video visit.

## Scenario 2: Digital Health Readiness and Telehealth

The hypothetical patient scenario shown in Textbox 2 reflects how passively collected data could link patients with digital health navigation services to improve digital health care outcomes.

Looking at Mr P’s case, he is a person who has ostensibly high digital health readiness through demonstrated skills, access to a network, and use of a health system app; however, he has also consistently had issues logging in for video visits, which adversely impacted his digital health care use and increased his risk of hospital readmissions. In this scenario, an automated alert based on previous patterns of digital health care use from electronic health record data triggered help with navigating video visits from a digital health navigator, which many health systems offer [36-38]. That alert could have triggered office staff to arrange an in-person appointment or home visit to assess his ability to use telehealth and provide help if he could not.

Having systematic processes in place to assess who is most appropriate for in-person versus remote or asynchronous care could guide efficient service delivery and use of resources. With their abundance of claims data and the opportunity to trial different variations of digital support pathways, integrated delivery and finance systems represent a unique setting where digital health readiness measures could be deployed, tested, and refined.

Digital health readiness assessments could be a key step toward making digital health implementation more systematic for all people, leading to greater equity and effectiveness. In many clinics, the process of selecting in-person care versus telemedicine could be tied to the nature of the medical issue, the judgment of scheduling and treating team members, and personal preferences (ie, a subset of patients who always want in-person care). Adding more specificity to digital health implementation through the creation of care delivery phenotypes—that is, providing navigation support for patients who are motivated to use digital health but are inexperienced—would optimize this care. It is likely that many opportunities for digital engagement and adoption of new tools are missed simply because health systems do not have robust ways to screen for who is best equipped and motivated for digital health but has not used it. Rather than limiting digital health to those patients who are already confident with technology, streamlined and methodical digital onboarding guided by a digital health readiness assessment could expand the reach of these tools to more patients. In turn, this could provide greater efficiency and, in some cases, reduced costs [39] for patients in scenarios where similar treatment outcomes have been achieved with video versus in-person visits [40]. Differentiating those who can complete a telemedicine appointment on their own from those who might need additional support would further expand digital health as a standard of care and improve the service experience for all patients.

To fully assess digital health readiness, we should also consider how a person’s situation may change over time as well as how personal and community resources could help them succeed. With an aging and increasingly medically complex population, digital health readiness phenotypes will likely be dynamic and may need to be repeated in certain circumstances, such as a major health event, functional decline, cognitive impairment, financial insecurity, or loss of family support [17]. In the event that a person can no longer use a particular tool, a support person may be best suited to provide digital health support in a convenient environment like a health center–affiliated or community-embedded internet clinic [41]. Furthermore, studies have shown that patients with limited technology experience are often able to complete a telehealth visit with the help of a family member, friend, or caregiver—thereby providing an opportunity to engage those with lower digital health readiness from the onset [42,43]. Partnering with patients, families, and communities could help to personalize digital care delivery pathways even further and improve engagement.

**Hypothetical patient scenario 2.**

Mr P is a man aged 75 years who has been hospitalized 5 times in the past year for decompensated heart failure. He has a smartphone that enables him to message his primary care provider and heart failure specialist via his health system’s patient portal. As he transitioned between hospitals, skilled nursing facilities, and home, he missed multiple follow-ups. His primary care office proactively contacts him at home and sets up a video visit to reestablish care. When the time for the appointment arrives, his primary care provider begins the visit but Mr P cannot log in. After he spends 10 minutes of the 30-minute appointment trying to use the videoconferencing platform, his doctor switches to a phone visit. At the end of the visit, his doctor receives an automated alert from the electronic health record noting that prior scheduled video visits have been converted to phone visits. Looking deeper into the situation, the doctor notices that recurrent telehealth platform issues have taken time away from health care providers to discuss all aspects of his health issues in prior visits—especially dietary counseling (a key reason for his hospitalizations). After the visit, Mr P is referred for an in-person digital health navigation session where he is instructed on ways to troubleshoot the telehealth platform and demonstrate that he can use the videoconferencing service independently.

## Challenges of Implementing Digital Health Readiness Assessments

While digital health readiness assessments apply to individual patients, health systems will also need to build infrastructure to respond to the results of these assessments in a meaningful way to realize their full potential. There are established standards to promote organizational health literacy within health systems that could be applied to digital health implementation—including fostering a culture among employees that promotes communication and engagement with patients and families using technology [44,45].

Moreover, HCPs and team members also have varying levels of digital health readiness that affect the implementation of digital health readiness assessments. Similar to medication prescribing, HCPs often serve as gatekeepers for recommending and promoting digital health tools. HCPs’ awareness and perceptions of the benefits of digital tools have been identified as determinants of mobile app uptake for chronic disease management [46]. While one might assume that HCPs would have more than adequate digital health readiness and literacy, some studies of hospitals in resource-limited settings worldwide (including one from Ethiopia during the COVID-19 pandemic) have found that less than half of HCPs had high digital health literacy [47]. Health care systems must consider the levels of technological awareness, comfort, and competence among their HCPs when considering more equitable digital health implementation.

There are also potential risks and ethical concerns involved in digital health implementation. With studies showing that digital health engagement is lower among older people, those who require an interpreter, and those who live in more deprived areas [48], efforts to shift more and more health care to digital platforms could exacerbate gaps in care. Furthermore, while the aforementioned evaluation frameworks for digital health tools do consider inclusivity and equity for diverse populations, studies have suggested that only 58% of mobile app evaluation frameworks do so, meaning that vital perspectives on technological tools may still be left behind [49]. Tying back to digital health literacy and health literacy, patients could experience delays in care if they were to choose telehealth or a patient portal message for a condition that warrants in-person evaluation. Personal health data collection and security are also important considerations for making sure that participating in digital health care is safe for all users.

A challenge of aptitude- and analytics-based digital health readiness assessment approaches is that they could amplify societal inequities if not designed carefully and evaluated among minoritized populations. Assessments based solely on aptitude may be biased against other-abled individuals with visual or hearing impairments or people whose primary language is not English. Moreover, given the complex array of factors that impact digital health engagement, digital health readiness assessments cannot be perfectly comprehensive. Digital health literacy is a single digital determinant of health that incorporates a person’s underlying literacy, numeracy, and general health literacy—each of which could not be measured or acted upon in a single clinic visit. Using passively collected data carries the risk of perpetuating systemic biases through algorithmic determinism (eg, the perpetuation of systemic bias through algorithms trained on biased data) [50,51] and underrepresentation of marginalized groups in data overall [52], which could further contribute to the digital health divide [21]. It will be important to test and validate digital health readiness assessments among diverse patients. If the evidence for these assessments has not yet been established among certain groups, this should be noted in the electronic health record and factored into how they are deployed and understood.

## Conclusion

Assessing and supporting individual patient-level digital health readiness is a crucial step toward maximizing benefits from digital health care and could provide a path toward greater digital health equity. More systematic approaches to support patients with low digital health readiness could ensure that assessments are actionable for clinicians, payors, and health systems. If we can work to increase the reach of health technology to keep up with the evolution of the consumer electronics market, more patients could be empowered to enter the digital health care age and benefit from these new tools.

## Abbreviations

CGM: continuous glucose monitor
CHAT: Conversational Health Literacy Assessment Tool
CMS: Center for Medicare and Medicaid Services
eHEALS: eHealth Literacy Scale
HCP: health care provider

## References

[1] Samson L, Tarazi W, Turrini G (2021). Medicare beneficiaries’ use of telehealth in 2020: trends by beneficiary characteristics and location. Office of the Assistant Secretary for Planning and Evaluation.

[2] Lee E, Grigorescu V, Enogieru I (2023). Updated national survey trends in telehealth utilization and modality (2021-2022). Office of the Assistant Secretary for Planning and Evaluation.

[3] Lee EY, Cha S, Yun J, Lim S, Lee J, Ahn Y, Yoon K, Hyun MK, Ko S (2022). Efficacy of personalized diabetes self-care using an electronic medical record-integrated mobile app in patients with type 2 diabetes: 6-month randomized controlled trial. J Med Internet Res.

[4] Sabharwal M, Misra A, Ghosh A, Chopra G (2022). Efficacy of digitally supported and real-time self-monitoring of blood glucose-driven counseling in patients with type 2 diabetes mellitus: a real-world, retrospective study in north India. Diabetes Metab Syndr Obes.

[5] Rollman BL, Herbeck Belnap Bea, Abebe KZ, Spring MB, Rotondi AJ, Rothenberger SD, Karp JF (2018). Effectiveness of online collaborative care for treating mood and anxiety disorders in primary care: a randomized clinical trial. JAMA Psychiatry.

[6] Li R, Liang N, Bu F, Hesketh T (2020). The effectiveness of self-management of hypertension in adults using mobile health: systematic review and meta-analysis. JMIR Mhealth Uhealth.

[7] Fanning J, Brooks AK, Hsieh KL, Kershner K, Furlipa J, Nicklas BJ, Rejeski WJ (2022). The effects of a pain management-focused mobile health behavior intervention on older adults' self-efficacy, satisfaction with functioning, and quality of life: a randomized pilot trial. Int J Behav Med.

[8] Lucas JW, Villarroel MA (2022). Telemedicine use among adults: United States, 2021. NCHS Data Brief.

[9] Bober T, Rothenberger S, Lin J, Ng JM, Zupa M (2023). Factors associated with receipt of diabetes self-management education and support for type 2 diabetes: potential for a population health management approach. J Diabetes Sci Technol.

[10] Reddick CG, Enriquez R, Harris RJ, Sharma B (2020). Determinants of broadband access and affordability: An analysis of a community survey on the digital divide. Cities.

[11] Norman CD, Skinner HA (2006). eHealth literacy: essential skills for consumer health in a networked world. J Med Internet Res.

[12] van der Vaart Rosalie, Drossaert C (2017). Development of the Digital Health Literacy Instrument: measuring a broad spectrum of health 1.0 and health 2.0 skills. J Med Internet Res.

[13] Norman CD, Skinner HA (2006). eHEALS: the eHealth Literacy Scale. J Med Internet Res.

[14] Richardson S, Lawrence K, Schoenthaler AM, Mann D (2022). A framework for digital health equity. NPJ Digit Med.

[15] Baker DW (2006). The meaning and the measure of health literacy. J Gen Intern Med.

[16] Pelikan JM (2014). The evolving concept of the health literate health care organization. Tervise Arengu Instituut.

[17] Karnoe A, Furstrand D, Christensen KB, Norgaard O, Kayser L (2018). Assessing competencies needed to engage with digital health services: development of the eHealth Literacy Assessment Toolkit. J Med Internet Res.

[18] Lennon MR, Bouamrane M, Devlin AM, O'Connor S, O'Donnell C, Chetty U, Agbakoba R, Bikker A, Grieve E, Finch T, Watson N, Wyke S, Mair FS (2017). Readiness for delivering digital health at scale: lessons from a longitudinal qualitative evaluation of a national digital health innovation program in the United Kingdom. J Med Internet Res.

[19] Getachew E, Woldeamanuel Y, Manyazewal T (2022). Capacity and readiness assessment of healthcare facilities for digital health interventions against tuberculosis and HIV in Addis Ababa, Ethiopia. Front Digit Health.

[20] Bingham JM, Rossi MA, Truong H (2022). Addressing the need for a telehealth readiness assessment tool as a digital health strategy. J Am Pharm Assoc (2003).

[21] 2024 Medicare Advantage and Part D Final Rule (CMS-4201-F). Centers for Medicare and Medicaid Services.

[22] Nelson LA, Pennings JS, Sommer EC, Popescu F, Barkin SL (2022). A 3-item measure of digital health care literacy: development and validation study. JMIR Form Res.

[23] Scherrenberg M, Falter M, Kaihara T, Xu L, van Leunen M, Kemps H, Kindermans H, Dendale P (2023). Development and validation of the Digital Health Readiness Questionnaire: prospective single-center survey study. J Med Internet Res.

[24] Kayser L, Karnoe A, Furstrand D, Batterham R, Christensen KB, Elsworth G, Osborne RH (2018). A multidimensional tool based on the eHealth Literacy Framework: development and initial validity testing of the eHealth Literacy Questionnaire (eHLQ). J Med Internet Res.

[25] Lyles C, Aguilera A, Nguyen O (2022). Bridging the digital health divide: how providers and plans can help communities better adopt digital health tools. California Health Care Foundation.

[26] Jaworski BK, Webb Hooper M, Aklin WM, Jean-Francois B, Elwood WN, Belis D, Riley WT, Hunter CM (2023). Advancing digital health equity: Directions for behavioral and social science research. Transl Behav Med.

[27] Miller-Rosales C, Morden NE, Brunette MF, Busch SH, Torous JB, Meara ER (2023). Provision of digital health technologies for opioid use disorder treatment by US health care organizations. JAMA Netw Open.

[28] Vijay A, Yancy CW (2024). Health equity in heart failure. Prog Cardiovasc Dis.

[29] Crump CA, Wernz C, Schlachta-Fairchild L, Steidle E, Duncan A, Cathers L (2021). Closing the digital health evidence gap: development of a predictive score to maximize patient outcomes. Telemed J E Health.

[30] Hughes H, Canino R, Sisson S (2021). A simple way to identify patients who need tech support for telemedicine. Harvard Business Review.

[31] NTIA Data Explorer. National Telecommunications and Information Administration.

[32] Yan X, Stults CD, Deng S, Liang S, Dillon E, Mudiganti S, Oscarson B, Jones JB, Frosch DL (2022). Do patients continue to use video visits? Factors related to continued video visit use. Popul Health Manag.

[33] Dang S, Muralidhar K, Li S, Tang F, Mintzer M, Ruiz J, Valencia WM (2022). Gap in willingness and access to video visit use among older high-risk veterans: cross-sectional study. J Med Internet Res.

[34] Chen J, Li KY, Andino J, Hill CE, Ng S, Steppe E, Ellimoottil C (2022). Predictors of audio-only versus video telehealth visits during the covid-19 pandemic. J Gen Intern Med.

[35] O'Hara Jonathan, Hawkins M, Batterham R, Dodson S, Osborne RH, Beauchamp A (2018). Conceptualisation and development of the Conversational Health Literacy Assessment Tool (CHAT). BMC Health Serv Res.

[36] Mechanic OJ, Lee EM, Sheehan HM, Dechen T, O'Donoghue AL, Anderson TS, Annas C, Harvey LB, Perkins AA, Severo MA, Stevens JP, Kimball AB (2022). Evaluation of telehealth visit attendance after implementation of a patient navigator program. JAMA Netw Open.

[37] Rodriguez JA, Charles J, Bates DW, Lyles C, Southworth B, Samal L (2023). Digital healthcare equity in primary care: implementing an integrated digital health navigator. J Am Med Inform Assoc.

[38] Perret S, Alon N, Carpenter-Song E, Myrick K, Thompson K, Li S, Sharma K, Torous J (2023). Standardising the role of a digital navigator in behavioural health: a systematic review. Lancet Digit Health.

[39] Patel KB, Turner K, Alishahi Tabriz A, Gonzalez BD, Oswald LB, Nguyen OT, Hong Y, Jim HSL, Nichols AC, Wang X, Robinson E, Naso C, Spiess PE (2023). Estimated indirect cost savings of using telehealth among nonelderly patients with cancer. JAMA Netw Open.

[40] Sammour Y, Main ML, Austin BA, Magalski A, Sperry BW (2022). Outpatient management of guideline-directed medical therapy for heart failure using telehealth: a comparison of in-office, video, and telephone visits. J Card Fail.

[41] Joseph N, Hider A, Contreras D (2023). Bridging the digital divide through on-site, health center–based internet clinics. NEJM Catal Innov Care Deliv.

[42] Chung GS, Ellimoottil CS, McCullough JS (2021). The role of social support in telehealth utilization among older adults in the United States during the COVID-19 pandemic. Telemed Rep.

[43] Semere W, Makaroun LK, Beach S, Schillinger D, Rosland A (2022). Family caregivers navigating the health care system: Evolving roles during the COVID-19 pandemic. Fam Syst Health.

[44] Brach C, Keller D, Hernandez L (2012). Ten attributes of health literate health care organizations. NAM Perspect.

[45] Brega AG, Hamer MK, Albright K, Brach C, Saliba D, Abbey D, Gritz RM (2019). Organizational health literacy: quality improvement measures with expert consensus. Health Lit Res Pract.

[46] Alaslawi H, Berrou I, Al Hamid A, Alhuwail D, Aslanpour Z (2022). Diabetes self-management apps: systematic review of adoption determinants and future research agenda. JMIR Diabetes.

[47] Ahmed MH, Guadie HA, Ngusie HS, Teferi GH, Gullslett MK, Hailegebreal S, Hunde MK, Donacho DO, Tilahun B, Siraj SS, Debele GR, Hajure M, Mengiste SA (2022). Digital health literacy during the COVID-19 pandemic among health care providers in resource-limited settings: cross-sectional study. JMIR Nurs.

[48] Chapman R, Haroon S, Simms-Williams N, Bhala N, Miah F, Nirantharakumar K, Ferguson J (2022). Socioeconomic deprivation, age and language are barriers to accessing personal health records: a cross-sectional study of a large hospital-based personal health record system. BMJ Open.

[49] Ramos G, Ponting C, Labao JP, Sobowale K (2021). Considerations of diversity, equity, and inclusion in mental health apps: A scoping review of evaluation frameworks. Behav Res Ther.

[50] Milossi M, Alexandropoulou-Egyptiadou E, Psannis KE (2021). AI ethics: algorithmic determinism or self-determination? The GPDR approach. IEEE Access.

[51] Cary MP, Zink A, Wei S, Olson A, Yan M, Senior R, Bessias S, Gadhoumi K, Jean-Pierre G, Wang D, Ledbetter LS, Economou-Zavlanos NJ, Obermeyer Z, Pencina MJ (2023). Mitigating racial and ethnic bias and advancing health equity in clinical algorithms: a scoping review. Health Aff (Millwood).

[52] Morey BN, Chang RC, Thomas KB, Tulua ', Penaia C, Tran VD, Pierson N, Greer JC, Bydalek M, Ponce N (2022). No equity without data equity: data reporting gaps for Native Hawaiians and Pacific Islanders as structural racism. J Health Polit Policy Law.

